# Early Life Low-Calorie Sweetener Consumption Impacts Energy Balance during Adulthood

**DOI:** 10.3390/nu14224709

**Published:** 2022-11-08

**Authors:** Anna M. R. Hayes, Linda Tsan, Alicia E. Kao, Grace M. Schwartz, Léa Décarie-Spain, Logan Tierno Lauer, Molly E. Klug, Lindsey A. Schier, Scott E. Kanoski

**Affiliations:** 1 Human and Evolutionary Biology Section, Department of Biological Sciences, University of Southern California, 3616 Trousdale Parkway, AHF-252, Los Angeles, CA 90089, USA; 2 Neuroscience Graduate Program, University of Southern California, Los Angeles, CA 90089, USA

**Keywords:** artificial sweeteners, non-nutritive sweeteners, sugar, Western diet, juvenile, adolescence, body weight, obesity, thermogenesis

## Abstract

Children frequently consume beverages that are either sweetened with sugars (sugar-sweetened beverages; SSB) or low-calorie sweeteners (LCS). Here, we evaluated the effects of habitual early life consumption of either SSB or LCS on energy balance later during adulthood. Male and female rats were provided with chow, water, and a solution containing either SSB (sucrose), LCS (acesulfame potassium (ACE-K) or stevia), or control (no solution) during the juvenile and adolescent periods (postnatal days 26–70). SSB or LCS consumption was voluntary and restricted within the recommended federal daily limits. When subsequently maintained on a cafeteria-style junk food diet (CAF; various high-fat, high-sugar foods) during adulthood, ACE-K-exposed rats demonstrated reduced caloric consumption vs. the controls, which contributed to lower body weights in female, but not male, ACE-K rats. These discrepant intakes and body weight effects in male ACE-K rats are likely to be based on reduced gene expression of thermogenic indicators (UCP1, BMP8B) in brown adipose tissue. Female stevia-exposed rats did not differ from the controls in terms of caloric intake or body weight, yet they consumed more SSB during CAF exposure in adulthood. None of the SSB-exposed rats, neither male nor female, differed from the controls in terms of total adult caloric consumption or body weight measures. The collective results reveal that early life LCS consumption alters sugar preference, body weight, and gene expression for markers of thermogenesis during adulthood, with both sex- and sweetener-dependent effects.

## 1. Introduction

Early life consumption of a Western diet is associated with increased caloric intake, susceptibility to obesity, and metabolic dysfunction during adulthood [1,2,3]. The underlying nutritive mechanisms mediating these effects, however, are poorly understood, given that established rodent obesogenic diet models vary from healthy control diets in two primary ways: (1) elevated dietary fat (particularly saturated fatty acids), and (2) elevated sugar levels. Given that children consume the most sugar of any age group, with sugar-sweetened beverages (SSBs) constituting a major source of sugar in their diet [4,5], it is imperative to understand the long-lasting implications of habitual early life sugar consumption, without concomitant elevated dietary fat, on eating patterns and body weight regulation during adulthood.

Based on the surging increase in obesity and nutrition-related co-morbidities among children and adolescents [6,7], increased focus has been placed on dietary and lifestyle interventions to better support healthy growth trajectories in early life [8,9]. One such strategy is to reduce sugar consumption. Decreasing sugar consumption while preserving pleasurable sweet taste in the diet can, at least theoretically, be achieved by consuming foods and beverages in which sugars have been fully or partially replaced with low-calorie sweeteners (LCSs); however, evidence from both humans and rodent models have been mixed and challenges the efficacy of using LCSs for weight management and energy balance maintenance [10,11,12,13]. Furthermore, LCS consumption has been shown to alter glucose homeostasis and gut peptide release in humans [14,15] and influences cephalic phase physiological signaling in rodent models [11,16]. Given that LCS consumption in children increased by ~200% between 1999 and 2012 [17], thus representing a societal shift towards caloric sugar replacement with LCSs during critical stages of development, it is important to mechanistically understand the long-term impacts of LCS consumption during early life on subsequent energy balance parameters during adulthood.

LCSs and caloric sugars bind to the same sweet receptors [18]; yet, unlike sugars, LCSs provide minimal to no energy to the body, and they comprise a wide variety of chemical structures [19]. Acesulfame potassium (ACE-K) is a commonly used synthetic LCS that retains its sweetness at high temperatures [20,21], thus making it an attractive LCS for use in a variety of processed and baked foods. Stevia, or the collection of Steviol glycosides, is extracted from the leaves of *Stevia rebaudiana* Bertoni, and it is one of the most popular ‘natural’ sweeteners, with recent exponential increase in its use in food and beverage products [20,22]. Although a number of studies have examined the effects of caloric sugars or LCSs on energy balance control [10,11,12,13,23], we emphasize two primary gaps in the literature: (1) the majority of mechanistic physiological rodent model research has used the LCS saccharin (e.g., [11,16,24,25,26,27]), which is not commonly consumed by humans; and (2) rodent model research often involves sugar or LCS consumption that is far in excess of what is consumed in human populations and involves involuntary consumption conditions where sugar or LCS is supplemented in drinking water. Therefore, in the present study, we aimed to fill critical gaps in the literature by assessing the long-term impacts of early life consumption of sugar or LCSs commonly consumed by humans (ACE-K or stevia) on eating behavior, metabolic outcomes, and body weight regulation during adulthood using a rat model wherein consumption was voluntary and restricted within federal recommended daily limits. To evaluate the potential impacts of early life sugar or LCS consumption on food choice during adulthood, our adult diet model employed an obesogenic cafeteria-style junk food diet (CAF) that involved a variety of food options high in dietary fat, sugar, or both.

## 2. Methods

### 2.1. Study Design

Male and female Sprague Dawley rats (postnatal day (PN) 25; 50–70 g) were purchased from Envigo (Indianapolis, IN, USA). Upon arrival, the juvenile rats were housed individually in a climate controlled (22–24 °C) environment with a 12:12 h light/dark cycle (lights off at 18:00). The rats were maintained on ad libitum standard rodent chow (Lab Diet 5001; PMI Nutrition International, Brentwood, MO, USA; 29.8% kcal from protein, 13.4% kcal from fat, 56.7% kcal from carbohydrate) and water. Between PN 26 and 70, a period that spans the juvenile stage, adolescence, and young adulthood [28], the rats received their experimental early life diets (Section 2.2) following randomization into groups of comparable weights. Body weights were recorded daily, whereas water consumption and chow intake were recorded 3 times per week. All procedures were approved by the Institutional Animal Care and Use Committee at the University of Southern California (protocol #21096).

### 2.2. Early Life Diets

Juvenile male and female rats (n = 9 per sex) were given daily amounts of acesulfame potassium (ACE-K group; catalog # A2815, Spectrum Chemical, Gardena, CA, USA; 0.1% ACE-K weight/volume (*w*/*v*) in reverse osmosis (RO) water; ~15 mg/kg), sucrose (10% KCAL SUG group; C&H Crockett, Crockett, CA, USA; 11% *w/v* sucrose in RO water), or stevia (STEVIA group; JG Group, Burlington, ON, Canada; 0.033% stevia *w/v* in RO water; ~4 mg/kg). The dose of ACE-K and stevia delivered daily was calculated to be within the U.S. FDA’s acceptable daily intake (ADI) of each sweetener according to body weight. Throughout the early life diet exposure period, the ACE-K and stevia concentrations were fixed, but the volumes administered were increased daily, and individually for each rat, in order to provide a fixed mg/kg dose across growth. LCSs were administered for voluntary consumption via a syringe into empty rodent sippers (part #ST7.0-BO with standard stoppers manufactured by Alternative Design Manufacturing & Supply, Siloam Springs, AR, USA). Each sipper had a vinyl cap (6 mm inner diameter, Jocon SF9000) attached to its end to prevent leakage, and it was placed on the wire rack adjacent to the rat’s chow and water bottle. The volume of sucrose delivered daily was based on general guidelines for sugar intake (<10% of total calories per day; Dietary Guidelines for Americans [29]) and was calculated to be equivalent to approximately 10% of the calories that were consumed from chow during the previous day for each individual rat. Sucrose was provided using a syringe to inject an exact volume into a 100 mL glass bottle containing a #6.5R rubber stopper and OT-100 straight 2.5″ sipper tube (Ancare, Bellmore, NY, USA). Voluntary consumption of the entire rations of ACE-K, sucrose, and stevia was verified daily by inspecting the tubes of all the animals. Each experimental group had its own control group (CTL; n = 9 per sex) that was given a tube filled with RO water at an equivalent volume as the experimental groups. The concentrations of ACE-K (0.1% *w/v*) and stevia (0.033% *w/v*) were determined based on our preliminary two bottle preference tests (concentrations preferred to water; data not shown) as well as concentrations used in published studies [30,31]. The concentration of sucrose (11% *w/v*) matched the concentration used in our previous studies [23,32,33,34], which was chosen to be comparable to the concentrations found in sugar-sweetened beverages that are commonly consumed by humans in modern Western cultures.

### 2.3. Glucose Tolerance Test (GTT)

Rats were habituated to oral gavage, food-restricted 22 h prior to the GTT as previously described [35], and water restricted the start of the dark cycle (15 h prior to the GTT). Five minutes prior to oral gavage with dextrose, baseline blood glucose readings were obtained from the tail tip and recorded using a blood glucose meter (One-touch Ultra2, LifeScan Inc., Milpitas, CA, USA). Each animal was then orally gavaged with dextrose in 0.9% sterile saline (20% *w/v*, 9.5 mL/kg by body weight), and tail tip blood glucose readings were obtained at 5, 10, 30, 60, and 120 min after gavage using a blood glucose meter.

### 2.4. Cafeteria Diet Access in Adulthood

At PN 82 (in adulthood), rats were transferred to hanging wire-bottom cages and were provided free access to a cafeteria (CAF) diet consisting of potato chips (Ruffles), chocolate peanut butter cups (Reese’s minis), 45% kcal high fat/sucrose chow (Research Diets D12451, New Brunswick, NJ, USA), a bottle of 11% weight/volume (*w/v*) high fructose corn syrup (HFCS)-55 solution (Best Flavors, Orange, CA, USA), and water. The concentration of sugar (11% *w/v*) was based on prior studies that aimed to model commonly consumed SSBs in humans [28,33]. In each home cage, there were individual, designated food hoppers for the accurate measurement of each solid food type. The potato chips and chocolate peanut butter cups were crushed or chopped to facilitate consumption through the hoppers. Papers were placed underneath the hanging wire cages to collect and weigh food spillage. Body weights and food intake were measured 3x/week during CAF diet access, and total (cumulative) kcal consumption, as well as the percentage of kcal consumption, of each of the CAF diet components were calculated.

### 2.5. Open Field

At PN 114, while on the CAF diet, the rats were tested in the open field task, a behavioral paradigm used in rodents to evaluate innate anxiety-like and locomotor activity behaviors [36]. The open field procedures were derived from Suarez et al. [37] and testing occurred during the light cycle. The apparatus consisted of a gray arena (60 cm × 56 cm), with a designated center zone within the arena (19 cm × 17.5 cm), placed under diffused lighting (30 lux) compared to the corners and edges (~18 lux). Animals were placed in the center of the box and allowed to freely explore for 10 min. The apparatus was cleaned with 10% ethanol between rats. Anxiety-like behavior was measured as time spent in the center zone, and locomotor activity was measured as distance travelled (m) in the apparatus during the task as quantified by a video tracking system (ANY-maze, Stoelting Co., Wood Dale, IL, USA).

### 2.6. Quantitative Polymerase Chain Reaction (qPCR)

Following an injection of an anesthetic (ketamine 90 mg/kg, xylazine 2.7 mg/kg, and acepromazine 0.64 mg/kg), interscapular brown adipose tissue (BAT) was dissected and flash frozen in a beaker filled with isopentane and surrounded by dry ice. To quantify the relative *UCP1* and *BMP8B* mRNA expression between the control (n = 18, 9 females and 9 males) and ACE-K (n = 18, 9 females and 9 males) rats in the BAT, a reverse transcriptase quantitative polymerase chain reaction (RT-qPCR) was performed as previously described [38]. Briefly, the total RNA was extracted from each sample using the RNeasy Lipid Tissue Mini Kit (Cat No. 74804, Qiagen, Hilden, Germany) according to the manufacturer’s instructions. The total RNA concentration per sample was measured using a NanoDrop Spectrophotometer (ND-ONE-W, ThermoFisher Scientific, Waltham, MA, USA). RNA (2000 ng) was reverse transcribed to cDNA using the QuantiTect Reverse Transcription Kit (Cat No. 205311, Qiagen) according to the manufacturer’s instructions. The following Applied Biosystems probes for use with rats were utilized: *Rplp0* (Rn03302271_gH), *Ucp1* (Rn00562126_m1), and *Bmp8b* (Rn01516089_gH). qPCR was performed with the Applied Biosystems TaqMan Fast Advanced Master Mix (Cat#4444557, ThermoFisher Scientific) using the Applied Biosystems QuantStudio 5 Real-Time PCR System (ThermoFisher Scientific). All reactions were conducted in triplicate, and control wells without a cDNA template were included in order to verify the absence of genomic DNA contamination. The triplicate Ct values for each sample were averaged and normalized to a *Rplp0* expression. The comparative 2^−ΔΔCt^ method was utilized in order to quantify relative expression levels between groups for the genes of interest.

### 2.7. Statistical Analysis 

Data are presented in all figures as mean ± standard errors (SEM) for error bars. The ACE-K, sugar, and stevia groups were analyzed with their respective control groups (but not compared to each other) because these groups were evaluated in separate experiments. Analyses were performed using Prism software (GraphPad Inc., version 8.4.2, San Diego, CA, USA), with significance considered at *p* < 0.05. Two-way analysis of variance (ANOVA) was used to assess the total caloric intake of standard chow, total caloric intake of the CAF diet, and body weight, employing Sidak’s multiple comparisons test when significant main effects were indicated. Regarding the caloric intake of standard chow, the total number of kcals consumed was assessed with factors of group (between-subjects), sex (between-subjects), and the group × sex interaction incorporated into the model. For caloric intake of the CAF diet, analyses were performed for the total number of kcals (with factors of group (between-subjects), sex (between-subjects), group × sex interaction) as well as the number of kcals consumed over time per sex (group (between-subjects), time (within-subjects), group × time interaction). Because significant main effects for sex were indicated for caloric intake of standard chow and CAF diet, separate analyses were performed for males and females for the other outcomes. Percentage caloric consumption of each of the CAF diet components over time was assessed separately for males and females using either repeated measures ANOVA or mixed effects analyses, with group (between-subjects, fixed), time (within-subjects, fixed), and the group × time interaction used as factors. Repeated measures ANOVA was performed for body weight analyses, with group (between-subjects), time (within-subjects), and the group × time interaction used as factors in the model. A mixed-effects analysis was used for glucose tolerance, with fixed effects of group, time, and the group × time interaction in the model and subject set as a random effect (thus making this a mixed-effects analysis). A two-tailed unpaired t-test was used to analyze outcomes from the open field behavioral task (anxiety-like behavior, locomotor activity) as well as the mRNA expression of *BMP8B* and *UCP1*.

## 3. Results

### 3.1. Early Life Habitual ACE-K Consumption Reduces the Total Caloric Intake of a Cafeteria Diet during Adulthood

**Figure 1:**
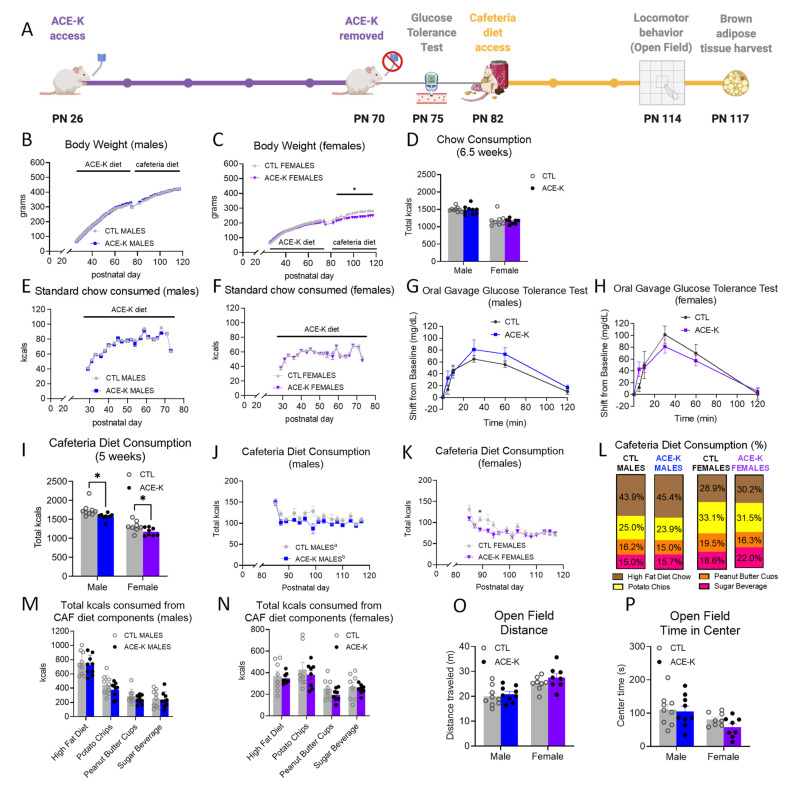
Effects of early life consumption of ACE-K on energy balance parameters during adulthood. Early life ACE-K consumption at the ADI (15 mg/kg; (**A**)) had no effect on body weight (**B**,**C**), standard chow intake (**D**–**F**), or glucose tolerance (**G**,**H**), relative to controls that did not have access to ACE-K. When challenged with a cafeteria diet in adulthood, both ACE-K males and females reduced their total caloric intake relative to controls (**I**–**K**), with no differences in kcals consumed from the cafeteria diet components (**L**–**N**). Although a reduced caloric intake decreased body weight in females (**C**), there were no differences in body weight in males (**B**). These results were not confounded by differences in locomotor activity (**O**) or anxiety-like behavior (**P**). Data are represented as mean ± SEM, * *p* < 0.05; different letters (a,b) indicate a significant main effect between diet groups in (**J**), *p* < 0.05. PN: postnatal day; CAF: cafeteria; CTL: control; ACE-K: acesulfame potassium; kcal: kilocalories.

During the ACE-K access period with maintenance on standard chow and water (PN 26-70; timeline shown in Figure 1A), neither body weights (Figure 1B,C), chow intake (Figure 1D–F), nor water consumption (Supplementary Figure S1) significantly differed between groups for either sex. At PN 75, after a brief washout period without ACE-K access, there were no differences in glucose tolerance between groups for either sex (Figure 1G,H). 

During the CAF access period (PN 82-117), male ACE-K rats had similar body weights compared to controls (Figure 1B), whereas female rats that consumed ACE-K during the early life period displayed a significant reduction in body weight (*p* < 0.05, Figure 1C). Both male and female rats previously exposed to ACE-K had significantly reduced overall caloric intake when maintained on the CAF diet (*p <* 0.05, Figure 1I–K). The overall reduction in calories consumed from the CAF diet over 5 weeks was not driven by differences in consumption of a particular component of the CAF diet (HFD, potato chips, peanut butter cups, and sugar beverage; Figure 1L–N and Supplementary Figures S1 and S4), and there were no differences in the macronutrient percentages consumed between groups (% kcals from fat, carbohydrates, protein; Supplementary Figure S4). Neither locomotor activity nor time spent in the center zone of the open field apparatus differed by group (Figure 1O,P). Collectively, results show that early life ACE-K consumption reduces caloric intake on a CAF diet during adulthood in both sexes, yet this caloric reduction was only accompanied by a reduction in body weight in female ACE-K rats.

### 3.2. Early Life Habitual Sugar Consumption Does Not Impact Cafeteria Diet Consumption in Adulthood

**Figure 2:**
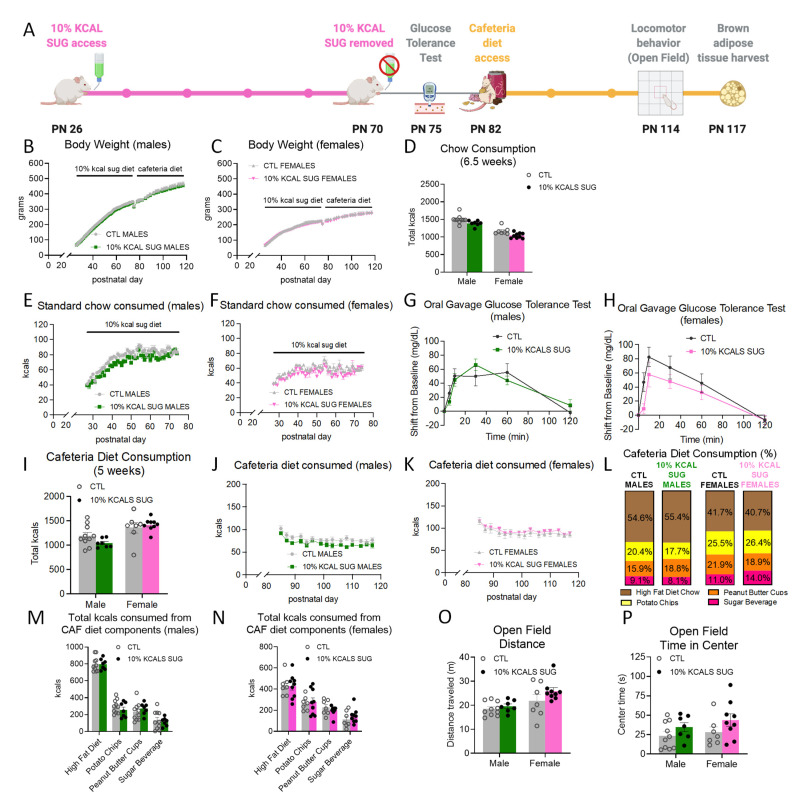
Effects of early life consumption of sugar at 10% of total calories consumed on energy balance parameters during adulthood. Limiting the consumption of sugar to 10% of the total calories consumed during the juvenile and adolescent periods (**A**) had no effect on body weight (**B**,**C**), chow intake (**D**–**F**), glucose tolerance (**G**,**H**), cafeteria diet consumption (**I**–**N**), locomotor activity (**O**), or anxiety-like behavior (**P**) relative to controls that did not have sugar access during adolescence. Data are represented as mean ± SEM; PN: postnatal day; CAF: cafeteria; CTL: control; SUG: sugar; kcal: kilocalories.

Early life access to a bottle of sucrose with a caloric content equivalent to 10% of the total number of calories consumed from chow the day before (from PN 26-70; timeline shown in Figure 2A) did not influence body weight relative to controls (Figure 2B,C). There were also no group differences in intake of chow (Figure 2D–F). Water intake decreased in male rats (significant main effect of group and the group × time interaction, with significant post hoc comparisons at PN 29 and 36, *p* < 0.05; Supplementary Figure S2) and female rats (significant group × time interaction, *p* < 0.05 no significant post hoc comparisons; Supplementary Figure S2). These differences in water intake were likely due to compensation for the volume of the sucrose beverage consumed per day (10–20 mL). At PN 75, after daily sucrose access ceased, there were no group differences in glucose tolerance (Figure 2G,H). The early life sucrose consumption also did not affect total caloric consumption of a cafeteria diet during adulthood, nor was the consumption of any of the individual dietary components impacted by group in either males or females (Figure 2I–N and Supplementary Figures S2 and S4). Likewise, locomotor activity (Figure 2O) and anxiety-like behavior (Figure 2P) were unaffected in the 10% KCAL SUG group.

### 3.3. Early Life Stevia Consumption Increases Sugar-Sweetened Beverage Consumption during Adult Cafeteria Diet Exposure

**Figure 3:**
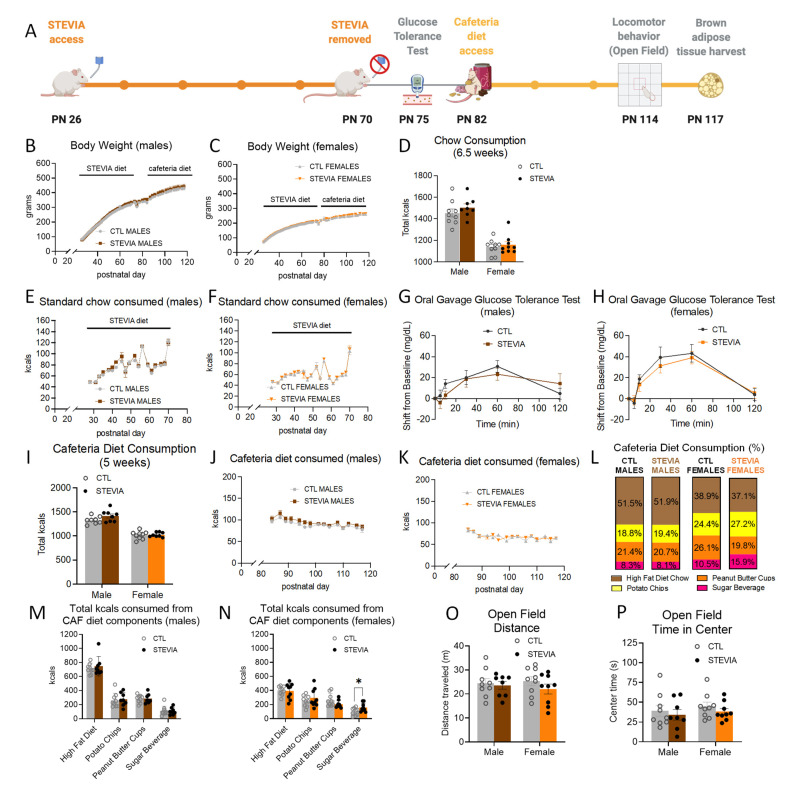
Effects of early life consumption of stevia in rats on energy balance parameters during adulthood. Early life stevia consumption at the ADI (4 mg/kg; (**A**)) had no effect on body weight (**B**,**C**), chow intake (**D**–**F**), glucose tolerance (**G**,**H**), or total calories consumed during adult exposure to the cafeteria diet (**I**–**K**) relative to controls that did not have sugar access during adolescence. There were no significant differences in the percentage of calories consumed from the four cafeteria diet components in either male or female rats (**L**). Early life stevia consumption did not alter the total calories consumed from the cafeteria diet components in male rats during adulthood (**M**), but it did significantly increase the number of calories consumed from the sugar beverage in female rats during cafeteria diet consumption in adulthood – in the absence of differences among the other three cafeteria diet components (**N**). Locomotor activity (**O**) and anxiety-like behavior (**P**) were not influenced by stevia exposure in early life. Data are represented as mean ± SEM, * *p* < 0.05; PN: postnatal day; CAF: cafeteria; CTL: control; kcal: kilocalories.

With daily stevia access from PN 26 and 70 dosed within the federal ADI levels (timeline shown in Figure 3A), body weights were not significantly different from the controls (Figure 3B,C). Neither males nor females in the stevia group displayed differences in intake of chow (Figure 3D–F) or water (Supplementary Figure S3) relative to the controls. At PN 75, following the cessation of daily stevia access, there were no group differences in glucose tolerance (Figure 3G,H). Although overall total caloric consumption in stevia-exposed males and females (Figure 3I–K) was not significantly different relative to controls, stevia-exposed females consumed significantly more of the sugar beverage relative to control females (~16% of total kcals consumed for stevia females vs. ~11% of total kcals consumed for CTL females; *p* < 0.05; Figure 3L,N, Supplementary Figure S3). Despite this increase in calories consumed from the sugar beverage, stevia-exposed female rats did not consume significantly more calories from carbohydrates relative to the controls (*p* = 0.11, Supplementary Figure S3). The consumption of other components of the cafeteria diet (HFD, potato chips, peanut butter cups) was not influenced by early life stevia exposure in females (Figure 3N and Supplementary Figures S3 and S4), and no differences in consumption of the cafeteria diet components were observed for stevia males (Figure 3L,M and Supplementary Figures S3 and S4). Similarly, locomotor activity (Figure 3O) and anxiety-like behavior (Figure 3P) were unaffected by early life stevia consumption followed by cafeteria diet consumption during adulthood. 

### 3.4. ACE-K Consumption during Early Life Reduces Brown Adipose Tissue Gene Expression of the Thermogenic Markers, BMP8B and UCP1, in Male Rats on a Cafeteria Diet

**Figure 4:**
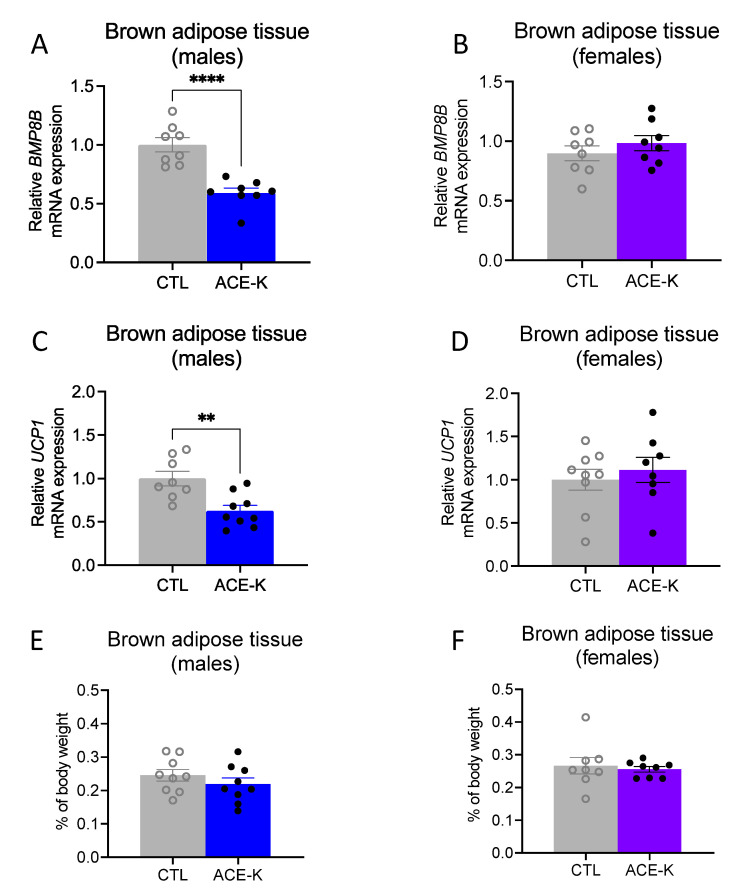
Effects of early life ACE-K consumption on adult brown adipose tissue (BAT) thermogenic markers. Early life ACE-K consumption at the ADI (15 mg/kg) decreased the relative mRNA expression of *BMP8B* and *UCP1* in male rats (**A**,**C**) relative to controls but had no effect in female rats (**B**,**D**). These effects were observed in the absence of differences in BAT percentage of total body weight for both sexes (**E**,**F**). Data are represented as mean ± SEM, ** *p* < 0.01, **** *p* < 0.0001; ACE-K: acesulfame potassium; CTL, control.

To further understand why early life ACE-K exposure reduces the consumption of a cafeteria diet in adulthood without a corresponding decrease in body weight in male rats (but not female rats, where both the caloric intake and body weighs were reduced), the interscapular BAT of ACE-K rats was analyzed for mRNA expression of *BMP8B* and *UCP1,* two common markers of BAT thermogenesis [39,40]. The mRNA levels of *BMP8B* were significantly reduced in ACE-K male rats relative to controls (*p* < 0.05, Figure 4A), but they were not significantly affected in ACE-K female rats (Figure 4B). Similarly, mRNA levels of *UCP1* were significantly reduced in ACE-K males relative to the control males (*p* < 0.05, Figure 4C), but they were not significantly affected in ACE-K female rats (Figure 4D). No differences in relative BAT weight (as percentage of body weight) were observed for either male or female ACE-K rats (Figure 4E,F). Thus, the discrepancy between caloric intake and body weight in male ACE-K rats vs. their controls could have been mediated by blunted thermogenesis and subsequent reduced energy expenditure.

## 4. Discussion 

The effects of habitual sugar or LCS consumption during early life periods of development on subsequent energy balance control during adulthood is poorly understood. Here, we reveal that early life voluntary consumption of a sucrose solution, modeling a concentration and daily caloric levels commonly consumed by humans, did not yield any effects on energy intake, body weight, or glucose homeostasis—including any impacts on energy intake and body weight when challenged with a cafeteria-style Western diet (CAF) diet during adulthood. For LCSs, early life habitual consumption of ACE-K, but not stevia, disrupted the energy balance upon adulthood consumption of a CAF diet, with notable sex-dependent effects. More specifically, both male and female ACE-K-exposed rats consumed fewer calories than controls during the adult CAF period, yet the females showed an accompanying significant reduction in body weight, whereas the males did not. This sex-dependent discrepancy in ACE-K rats was likely driven by the reduced mRNA expression of *BMP8B* and *UCP1* in brown adipose tissue (BAT) in males, whereas no differences were observed in these markers of thermogenic activity in females. 

The absence of effects resulting from early life sugar consumption on energy balance parameters was surprising, especially noting the ‘developmental programming hypothesis’, which posits that early life is a critical period for programming energy balance and metabolic health later in life [41]. However, previous research has indicated that adolescent consumption of either ad libitum or 5% kcals from sugar of comparable sugar solutions (HFCS or sucrose) in rats did not impart changes in caloric intake or body weight during early adulthood, despite resulting in long-lasting memory impairments and altered microbiome [23,32,33,34]. Present results, together with these previous findings, suggest that early life sugar consumption may have more profound long-term influences on memory function than on ingestive and metabolic outcomes.

Previous research has identified sex-specific effects of 4 weeks of adult ACE-K consumption on body weight of adult mice, such that males had increased body weights compared to controls, and females did not differ from the controls [42]. These differences were related to changes in gut bacteria community composition and functional genes related to energy metabolism. Our recent work using the early life LCS model revealed no substantial differences in the gut microbiome [43], but this previous study did not include CAF diet consumption during adulthood. Although both our present study and the work by Bian et al. [42] support the idea that the dysregulation of the energy balance is related to ACE-K consumption, and is more pronounced in males, there are several important factors to resolve that may be related to the discrepant findings between these two studies: rodent age, duration of exposure to ACE-K (PN 56-84 vs. PN 26-70), dose of ACE-K administered (37.5 mg/kg vs. ~15 mg/kg), route of administration (oral gavage vs. voluntary consumption in supplemental drinking water), and subsequent exposure to a Western/CAF diet. Nevertheless, our current findings provide a clear connection between early life ACE-K exposure within the federal recommended level), Western diet consumption in adulthood, and altered gene expression of markers for thermogenesis, thus revealing an LCS-induced offset in metabolism that is specific to males. 

A particularly notable finding from the present work is the effect of early life stevia consumption, with a significant increase in intake of the sugary beverage observed for females during adult CAF diet exposure compared to controls. However, the overall caloric intake for stevia-exposed females did not differ from controls, and no differences in any other outcomes were indicated for stevia-exposed males. Previous findings show that high doses of stevia (25, 250, 500, and 1000 mg/kg) result in body weight reductions beginning 6 weeks after exposure in adult female rats [44], which suggests that a longer period of exposure and a higher dose than the ADI of 4 mg/kg may be required in order to observe effects on energy balance for this particular LCS. Similar weight-reducing and notable anti-diabetic properties have been observed with high doses of stevia in diabetic rats [45]. In vitro work has further suggested that stevia may be an endocrine disruptor [46], but this effect has not been documented in humans or rodent models to date, to our knowledge. Regardless, our data indicate that, when restricted within federal recommended daily limits, the consumption of stevia during early life had no impact on overall energy balance yet increased consumption of sugary beverages during adulthood in a sex-dependent manner. The mechanisms underlying these results warrant further investigation. We previously revealed that early life LCS (ACE-K) consumption using a similar model to the current study reduced lingual sweet taste receptor expression, and that LCS consumption, independent of sweetener (ACE-K, stevia, or saccharin) or sex, increased the consumption of a sugar solution when offered alongside ad libitum healthy chow [43]. These previous findings, taken together with the present results, collectively indicate that the consumption of LCS in early life appears to have long-lasting effects on the voluntary consumption of sugary beverages during adulthood, although it is worth noting that no such outcomes were observed in the present study following ACE-K consumption. Yet, in the present study, food options beyond a sugar solution and healthy chow were presented during the adulthood CAF diet exposure, which could have influenced the overall sugar intake.

The distinct effects of early life exposure to ACE-K compared to stevia on adult energy balance parameters are noteworthy, especially considering the varying chemical structures of ACE-K and stevia and their synthetic vs. ‘natural’ origins. ACE-K is rapidly and completely absorbed into the body, yet it is not metabolized prior to being excreted, which occurs primarily through urine, within approximately 24 h after consumption [47,48,49]. Additionally, it is unknown if, or to what extent, the potassium salt dissociates from the acesulfame anion in ACE-K, and therefore ACE-K consumption could also alter circulating potassium levels, which may affect the normal function of the sodium–potassium pump and the ensuing hydrolysis of ATP to generate energy [47,50]. In contrast, stevia (i.e., a mix of steviol glycosides) is more slowly absorbed into the body and is specifically metabolized by colonic bacteria into the common metabolites steviol, or steviol glucuronide before being excreted, predominantly in feces [47,51,52]. Although the distinct means of the absorption, metabolism, and excretion of ACE-K and stevia are generally comparable in rat models vs. humans, a notable difference is that, in humans, stevia is predominantly excreted in urine instead of feces [47,51]. In light of these differences between ACE-K and stevia, our current findings—that ACE-K affected energy balance whereas stevia did not—suggest that the rate at which LCSs are absorbed into the body, as well as their metabolism and excretion, may be critical distinguishing features in terms of their role on body weight regulation.

Our findings for sex-specific differences in BAT gene expression of markers for thermogenesis during the adult Western diet period for rats after early life ACE-K consumption warrant further investigation. Notably, the estrous cycle has been linked to minor changes in the expression of *UCP1* mRNA in BAT and plasma leptin levels, whereas in ovariectomized rats no such links were observed [53]. Given our results of blunted mRNA expression of *UCP1* and *BMP8B* in males only, it is possible that the estrous cycle had a protective effect in females to prevent such perturbations. Furthermore, previous evidence revealed that ACE-K consumption increases insulin and leptin levels [54], which are metabolic hormones with distinct energetic effects in males vs. females [55]. It is possible that slight alterations in insulin and leptin signaling associated with ACE-K consumption could have contributed to the differences in gene expression of markers for thermogenesis observed between males and females in the present study. Although we did not measure insulin and leptin, these may be important systems to examine in future work, especially noting the body of evidence indicating that LCSs can disrupt glucose homeostasis [16,43,56].

The primary focus of the present research was to evaluate the impact of early life sweetener consumption (caloric and noncaloric) within federally recommended levels on subsequent food intake, body weight, and select metabolic measures (i.e., glucose tolerance and mRNA expression of *BMP8B* and *UCP1*). Additional insights could be gained from future studies focusing more directly on measures of metabolic signaling, including protein levels of thermogenic markers; serum levels of metabolic hormones such as insulin, leptin, and glucagon; serum levels of free-fatty acids and triglycerides; and tolerance tests for insulin and pyruvate. Although we did not track the estrous cycle in female rats, nor measure reproductive hormones, in light of our sex-specific findings, these measures would be beneficial in future research. Furthermore, given that our experimental objective was to test sweetener consumption within federally recommended limits, future work could instead utilize concentrations of ACE-K, sugar, and stevia that are matched for sweetness and/or palatability.

Collectively, our findings add further evidence challenging the efficacy of using LCSs for weight management and energy balance control. While early life ACE-K consumption was associated with reduced caloric intake during adulthood, this was accompanied by blunted expression of BAT thermogenic markers that promote energy expenditure, thus resulting in a net zero effect on body weight relative to controls. Importantly, our experiments involved the voluntary consumption of LCSs, which were restricted in accordance with the recommended federal daily limits, thus making the results more readily relevant to humans compared to the bulk of previous rodent LCS research that utilized excessive and involuntary consumption. These results further highlight the importance of sex differences in terms of the impacts of LCS consumption on energy balance, and they distinguish early life exposure as a critical period for lasting metabolic effects.

## 5. Conclusions

Our results reveal that habitual consumption of low-calorie sweeteners during early life development influences energy balance outcomes during adulthood, such that ACE-K consumption was linked with blunted expression of genes related to thermogenesis during adulthood in males, and stevia consumption was linked with elevated sugary beverage consumption during adulthood in females.

## Data Availability

The data supporting the findings of this research are available from the corresponding author (S.E.K.) upon reasonable request.

## Supplementary Materials

The following supporting information can be downloaded at: https://www.mdpi.com/article/10.3390/nu14224709/s1, Supplementary Figure S1: Expanded analyses of water intake and cafeteria diet consumption over time for early life ACE-K-exposed rats, Supplementary Figure S2: Expanded analyses of water intake and cafeteria diet consumption over time for early life sugar-exposed (10% kcal) rats, Supplementary Figure S3: Expanded analyses of water intake and cafeteria diet consumption over time for early life stevia-exposed rats, Supplementary Figure S4: Percentage of total calories consumed from cafeteria diet components during adult exposure.
