# Supplementary material for: Early Life Low-Calorie Sweetener Consumption Impacts Energy Balance during Adulthood
Source: Nutrients. 2022 Nov 8;14(22):4709. doi: 10.3390/nu14224709 (PMC9694170; doi:10.3390/nu14224709)
Supplement: Supplementary file 1 [file nutrients-14-04709-s001.zip › nutrients-2018042-supplementary.pdf]

Supplementary Figure S1

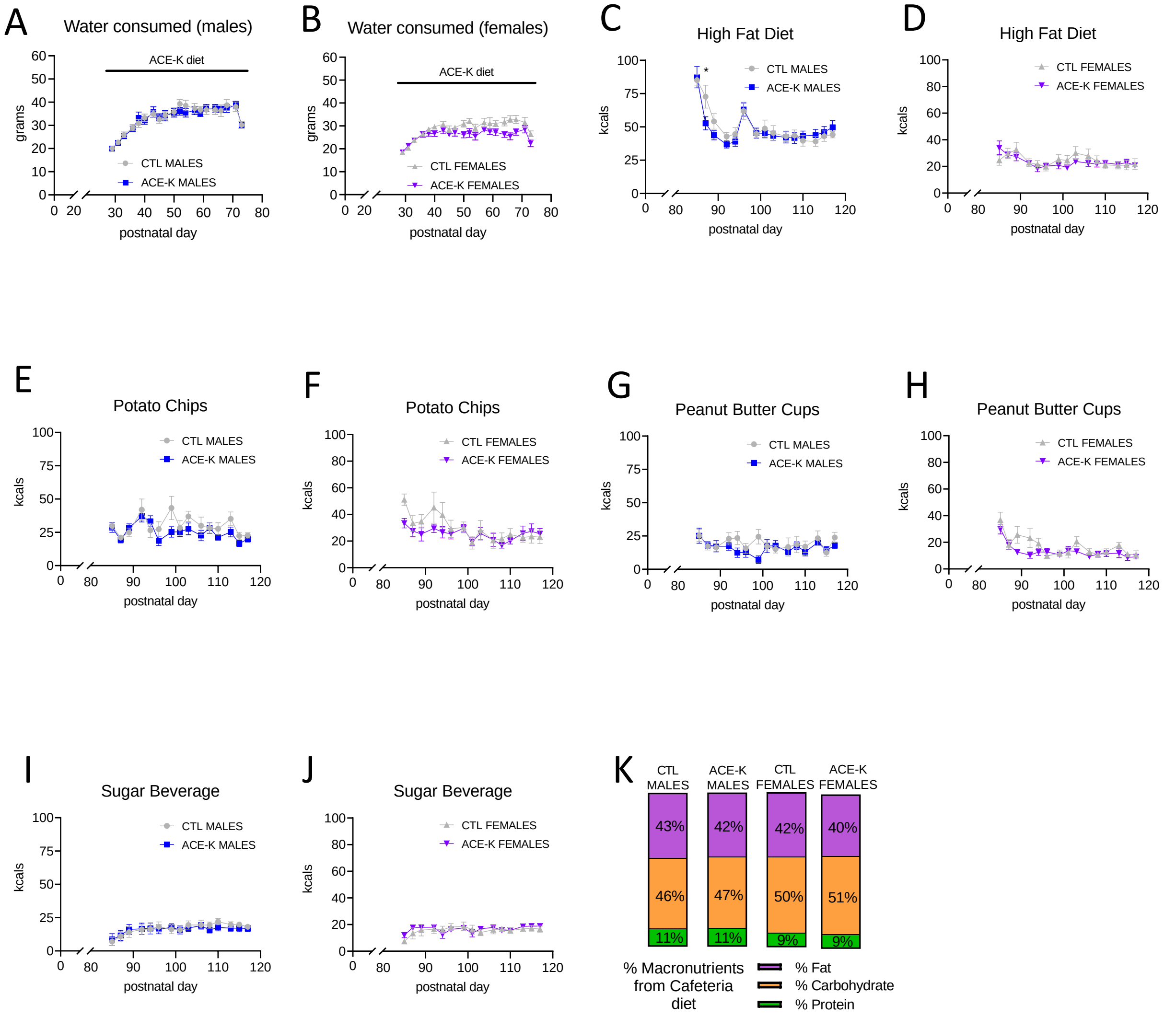

Supplementary Figure S1: Expanded analyses of water intake and cafeteria diet consumption over time for early life ACE-K-exposed rats. Early life acesulfame potassium (ACE-K) consumption had no effect on water intake in male or female rats (A-B). During the cafeteria (CAF) diet period in adulthood, there was a group × time interaction ($p = 0.02$) for total kcals consumed from high fat diet over time in males such that ACE-K males consumed fewer kcals from the high fat diet than control (CTL) males on postnatal day (PN) 84 (C). There were no differences in total kcals consumed over time from any of the other CAF diet components for males (potato chips, peanut butter cups, sugar beverage) and for any of the CAF diet components for females (D-J). Overall percentage of kcals consumed from fat, carbohydrate, and protein during the CAF diet period did not differ between ACE-K and CTL males or females (K). Data are represented as mean ± SEM, *$p < 0.05$; PN: postnatal day; CAF: cafeteria; CTL: control; ACE-K: acesulfame potassium; kcal: kilocalories.

Supplementary Figure S2

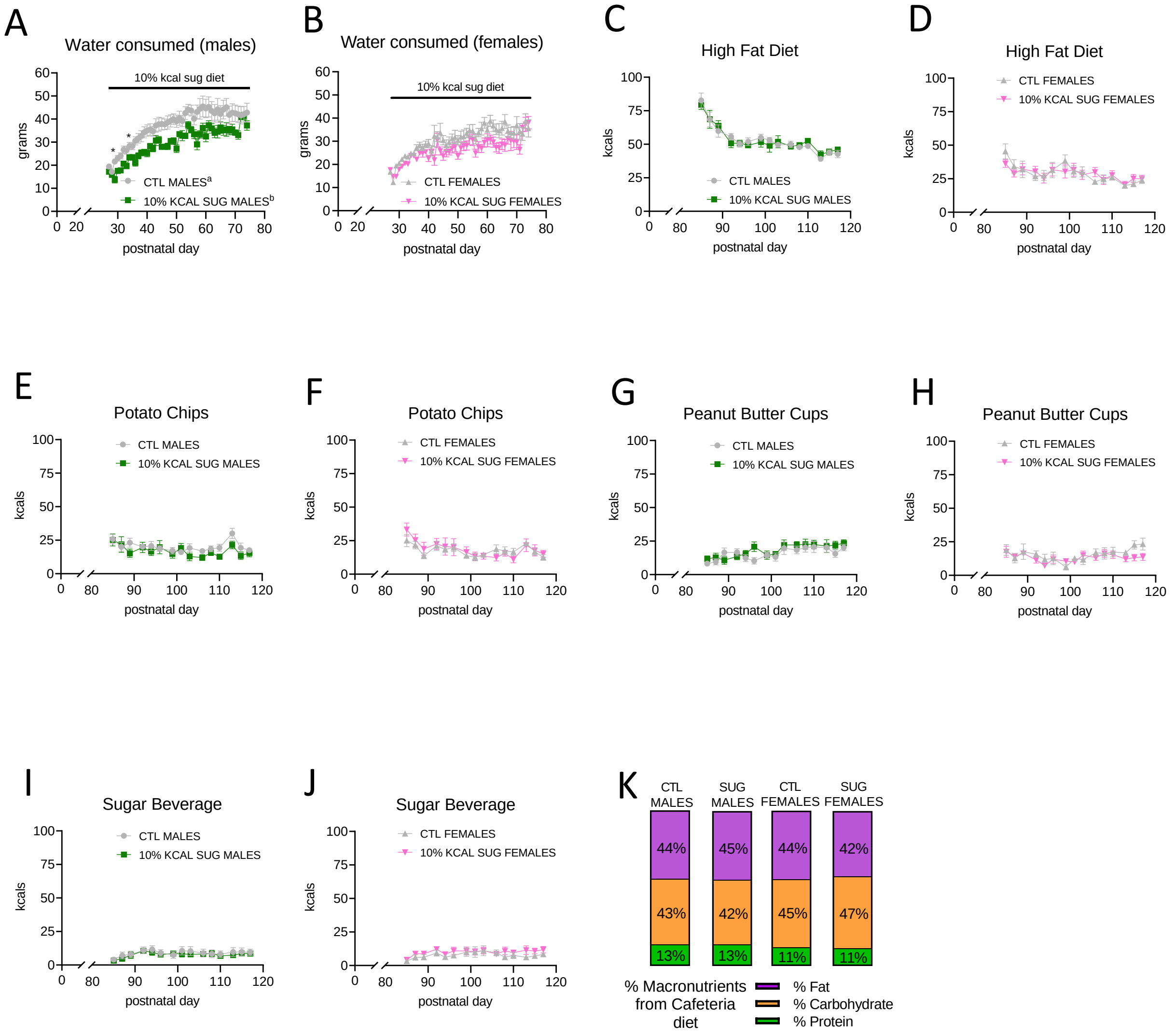

Supplementary Figure S2: Expanded analyses of water intake and cafeteria diet consumption over time for early life sugar-exposed (10% kcal) rats. Limiting consumption of sugar to 10% of total calories consumed during adolescence (10% KCAL SUG) decreased water intake in male rats (main effect of group [$p = 0.04$] and the group × time interaction [$p = 0.005$], with significant post hoc comparisons at PN 29 and 36; A) and female rats (group × time interaction [$p = 0.0004$], no significant post hoc comparisons; B). These differences in water intake are likely due to compensation for the volume of sucrose beverage provided per day (10-20 mL). During the cafeteria diet period in adulthood, there were no differences in total kcals consumed over time from any of the cafeteria diet components (high fat diet, potato chips, peanut butter cups, sugar beverage) for males or females (C-J). Overall percentage of kcals consumed from fat, carbohydrate, and protein during the CAF diet period did not differ between 10% KCAL SUG and CTL males or females (K). Data are represented as mean ± SEM, *$p < 0.05$, a significant main effect for diet group is indicated by different superscript letters accompanying group names (A); PN: postnatal day; CAF: cafeteria; CTL: control; SUG: sugar; kcal: kilocalories.

Supplementary Figure S3

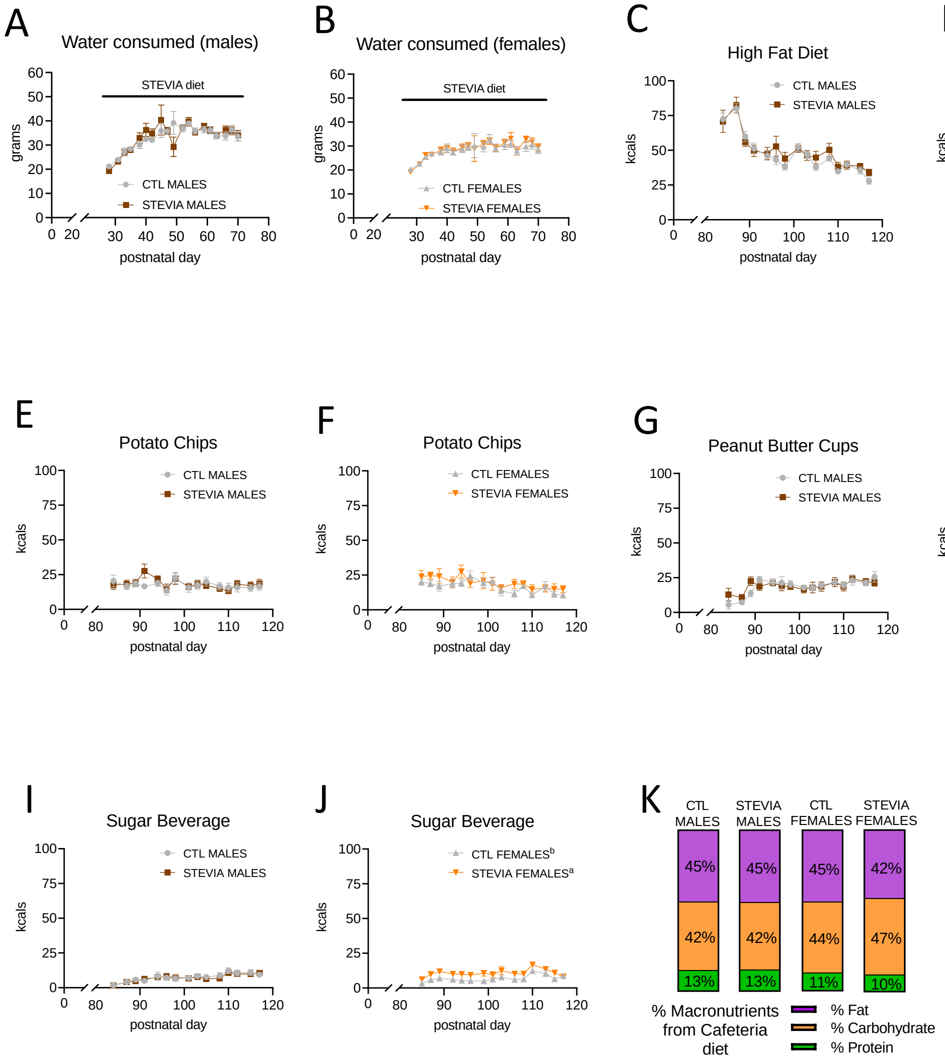

Supplementary Figure S3: Expanded analyses of water intake and cafeteria diet consumption over time for early life stevia-exposed rats. Early life stevia consumption (STEVIA) had no effect on water intake in male or female rats (A-B). During the cafeteria diet period in adulthood, there were no differences in total kcals consumed over time from the high fat diet, potato chips, or peanut butter cups for males or females (C-H). However, although there was no difference in total kcals consumed over time from the sugar beverage for male STEVIA rats (I), female STEVIA rats consumed more total kcals over time from the sugar beverage than control females (J). Overall percentage of kcals consumed from fat, carbohydrate, and protein during the CAF diet period did not differ between STEVIA and CTL males or females (K). Data are represented as mean ± SEM, $*p < 0.05$, a significant main effect for diet group is indicated by different superscript letters accompanying group names (J); PN: postnatal day; CAF: cafeteria; CTL: control; kcal: kilocalories.

Supplementary Figure S4

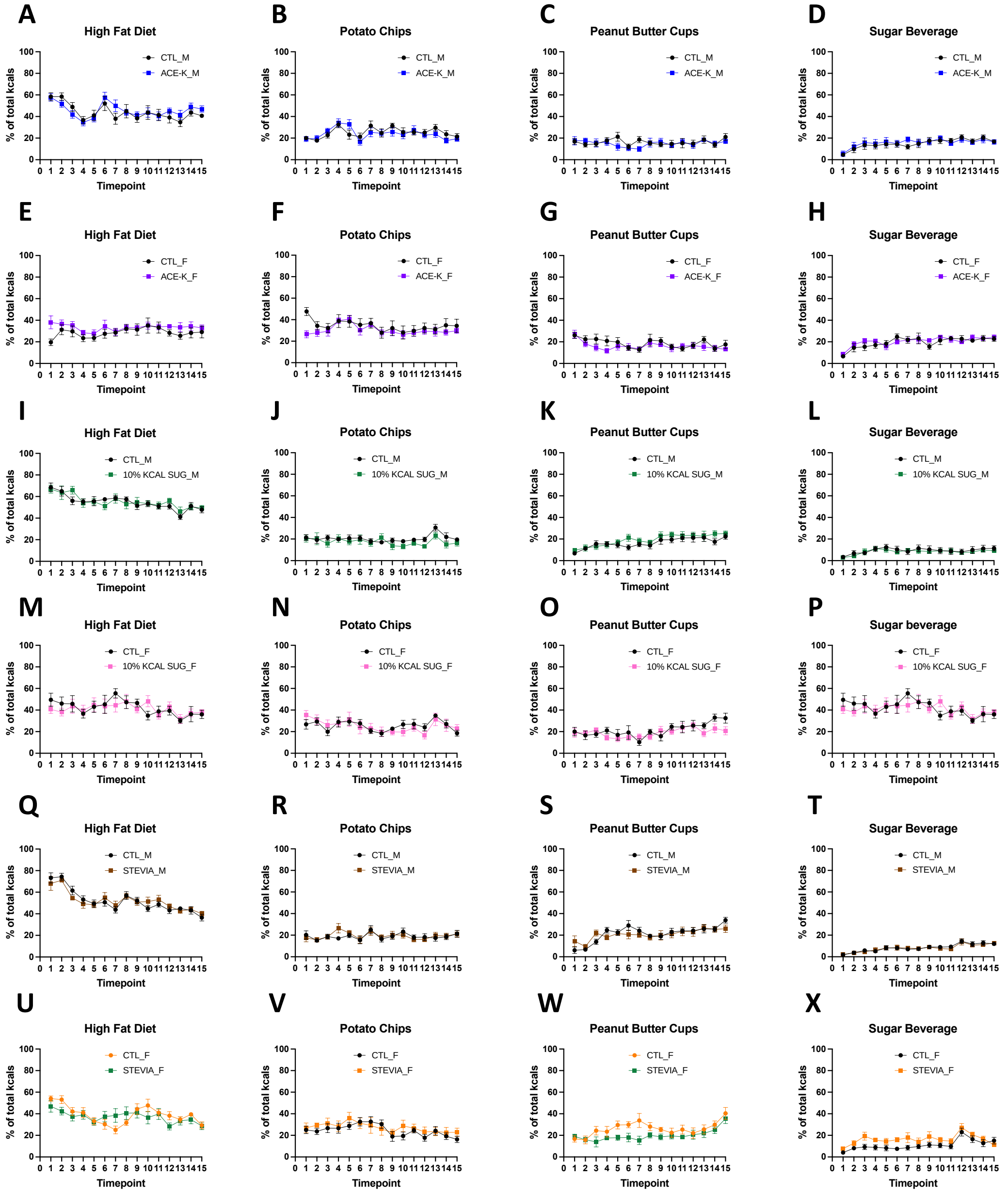

Supplementary Figure S4: Percentage of total calories consumed from cafeteria diet components during adult exposure. When examined as percentage of total calories consumed from each of the cafeteria diet components (% total kcals from high fat diet, potato chips, peanut butter cups, or sugar beverage), there were no differences for ACE-K males (vs. control males; A-D), ACE-K females (vs. control females; E-H), 10% KCAL SUG males (vs. control males; I-L), 10% KCAL SUG females (vs. control females, M-P), STEVIA males (vs. control males; Q-T), or STEVIA females (vs. control females; U-X). Data are represented as mean ± SEM; PN: postnatal day; CTL: control; kcal: kilocalories.
